# Protective effect of bone marrow mesenchymal stem cells modified with klotho on renal ischemia-reperfusion injury

**DOI:** 10.1080/0886022X.2019.1588131

**Published:** 2019-04-03

**Authors:** Li-Bo Xie, Xi Chen, Bo Chen, Xian-Ding Wang, Rui Jiang, Yi-Ping Lu

**Affiliations:** a Department of Urology, The Affiliated Hospital of Southwest Medical University, Luzhou, China;; b School of Biology Science, East China Normal University, Shanghai, China;; c Department of Human Anatomy, Southwest Medical University, Luzhou, China;; d Department of Urology, The Institution of Urology, West China Hospital, Sichuan University, Chengdu, China

**Keywords:** Renal ischemia-reperfusion injury, bone marrow mesenchymal stem cells, gene transfection, Klotho, FoxO1

## Abstract

**Objective:** To detect the combination protective effect of bone marrow mesenchymal stem cells (BMSCs) and Klotho gene on the renal ischemia-reperfusion injury (RIRI).

**Methods:** BMSCs isolated from rats were transfected with Klotho gene to form BMSC^Kl^. We injected BMSC^Kl^ to allogenic rat RIRI model. After 24 h and 72 h, we detected the serum creatinine (SCr), malondialdehyde (MDA), and superoxide dismutase (SOD) in renal tissue, Hematoxylin-eosin (HE) staining, and TUNEL of renal pathology. The expression of FoxO1 and p-FoxO1 in post-hypoxia tubular epithelial cells of normal rat kidney (NRK-52E) were detected by Western blot after cocultured with BMSC^Kl^.

**Results:** Comparing with BMSC^Con^ group, Rats in BMSC^Kl^ group had lower SCr and MDA but higher SOD. Both HE and TUNEL score of renal tissue in BMSC^Kl^ group were lower than that of BMSC^Con^ group. Western blot indicated that FoxO1 was upregulated, while p-FoxO1 was downregulated in post-hypoxia NRK-52E cells.

**Conclusions:** BMSCs transfected with Klotho gene can further ameliorate RIRI. The possible mechanism may be attributed to the upregulation of SOD in NRK-52E caused by Klotho-FoxO1 axis.

## Introduction

Renal ischemia-reperfusion injury (RIRI) is a common pathological process in patients with renal tumor who need nephron-sparing surgery [1]. In kidney transplantation, ischemia time of renal allograft is longer, so RIRI is more severe. RIRI can not only cause delayed graft function [2], but also stimulate humoral immunity so as to increase the risk of acute rejection, which could seriously affect allograft function and survival. Therefore, seeking for a strategy to relieve RIRI had been the key point of research in the field of solid organ transplantation.

Bone marrow mesenchymal stem cells (BMSCs) have the characteristics of multilineage differentiation, hematopoietic support, and self-replication. In addition, BMSCs have the function of immunomodulatory, anti-inflammatory and tissue renovation. The main pathological manifestation of RIRI was inflammatory reaction, which would decrease the sensibility of kidney microcirculation to vasodilators. With the ability of inflammatory chemotaxis, BMSCs was expected to become an ideal preparation to alleviate RIRI related inflammation. Compared to bone marrow hematopoietic stem cells, BMSCs have poor differentiation but stronger paracrine function, which were found to be just the main mechanism to relieve RIRI. In the treatment of myocardial infarction, some clinical researches have confirmed the repair function of BMSCs [3].

However, in the field of RIRI, limited effect of BMSCs was observed [4]. Improving BMSCs for better effect to RIRI is a promising research with ambitious clinical prospects. Meanwhile, Klotho gene has been found to have a protective effect on RIRI [5], but there is still no report of the combined effection of BMSCs and Klotho in RIRI treatment. Gene engineering technology could give BMSCs new secretion characteristics, which would enhance BMSCs paracrine ability in order to improve the repair effect of RIRI.

In this study, we engineered rat BMSCs to overexpress Klotho and injected the modifed BMSCs into RIRI model rats via left carotid artery. To our knowledge, this was the first study to investigate whether BMSCs delivery of Klotho attenuates RIRI.

## Methods

### Isolation, differentiation, and immunophenotyping of BMSCs

BMSCs were obtained and cultured from 2-week-old male Sprague Dawley (SD) rats. The animal experiments were approved through institutional ethics committee (Approval number: 20160010).

Tibia and femur of SD rat was flushed with medium of low glucose Dulbecco’s modified eagle medium (L-DMEM). After centrifugation, BMSCs were cultured in L-DMEM with 10% fetal bovine serum (FBS). When BMSCs grew to the third generation, we identified them through osteogenic and adipogenic induction, and cell suface markers (CD29, CD34, CD45, and CD90) using flow cytometry analysis. BMSCs were respectively transfected with green fluorescent protein (GFP) adenovirus and Klotho-GFP adenovirus to form BMSC^Con^ (BMSCs transfected with GFP adenovirus) and BMSC^Kl^ (BMSCs transfected with Klotho-GFP adenovirus). The expression of Klotho protein was detected by Western blot and enzyme-linked immunosorbent assay (ELISA). BMSCs proliferation after transfection was detected by CCK-8 (Institution of Tongren chemistry, Japan).

### Coculture model with NRK-52E cells

As specific rat renal proximal tubular cell line, NRK-52E (Kindly given by Shengfu Li, Transplantation immune laboratory, Sichuan University) was used to set up the coculture model by using a Transwell assay (pore size, 0.4 µm; Costar^®^; Corning Life Science, Corning, NY). NRK-52E cells were firstly implanted in six-well dishes filled with RPMI-1640 and 10% FBS under anoxic environment (94%N2 + 1%02 + 5%CO2). After 24 h, we changed its medium (also RPMI-1640 with 10%FBS) and put the cells into aerobic environment (95% 02 + 5% CO2) for 12 h. At the beginning of reoxygenation, cells were divided into three groups. NRK-52E cells in group 1 were cultured without any treatment, while NRK-52E cells in group 2 were treated by BMSC^Con^ (2 × 10^5^). NRK-52E cells in group 3 were treated by BMSC^Kl^ (2 × 10^5^) in the same conditions as group 2. BMSC^Con^ or BMSC^Kl^ were cocultured into the upper room of NRK-52E cells for 48 h. Then the expression levels of forkhead box protein O1 (FoxO1) and phosphorylated FoxO1 (p-FoxO1) in cocultured NRK-52E cells were detected by Western Blot analysis, which was used to identify the antiapoptotic mechanisms of BMSC^Kl^.

### Rat model of RIRI

The 10-week old allogenic male SD rats were used to form RIRI model. They were anesthetized with 10% hydral (3 mL/kg) through abdominal cavity injection. The right nephrectomy was performed for all the experiment rats. In the sham group, we just separated the left renal pedicle without blocking it. However, in the other three groups, the left renal pedicle was clamped with atraumatic vascular clamps for 45 min. Phosphate buffered saline (PBS) group was given with 1 mL PBS through left carotid artery after open clip. BMSC^Con^ group and BMSC^Kl^ group were separately injected with 1 mL of PBS diluted with BMSC^Con^ (1 × 10^6^/ml) and BMSC^Kl^ (1 × 10^6^/ml). We have ensured the blood recovery according to the color restoration of kidney. Then we closed the abdominal cavity and gave them normal diet after palinesthesia. Rats in each group (*n* = 12) were divided into two subgroups (*n* = 6, sacrificed respectively at 24 h and 72 h after operation). Half of the left kidney were weighted, homogenated, and centrifugated to detect superoxide dismutase (SOD) and malondialdehyde (MDA) in renal tissue. Whole blood (2 mL) of the inferior vena cava was taken for determination of serum creatinine (SCr).

Hematoxylin-eosin (HE) staining was made using routine pathological technique, and its slice was single-blindly read by two pathologists. The proportion of renal damage (tubular epithelial swelling, vacuolar degeneration, necrosis, and desquamation) was graded, and a semiquantitative score of tubular damage was determined as follows: 0: no damage; 1: 10%; 2: 11–25%; 3: 26–45%; 4: 46–75%; 5: >75% [6]. Apoptosis ratio of renal tubular epithelial cells was detected by terminal dexynucleotidyl transferase-mediated dUTP nick end labeling (TUNEL) method. The proportion of apoptosis was calculated as follows: Apoptosis Index (AI) = Total number of apoptotic cells/Total observation cell number × 100%.

### Statistical methods

All the data were processed by SPSS 19 (Chicago, IL) software and expressed as Mean with Standard Deviation (*x*±SD). Differences among groups were described with GraphPad Prism (GraphPad Software, La Jolla, CA). The ANOVA test of independent samples was used to compare the two groups and *p* < 0.05 was defined as statistical significance.

## Result

### Identification of BMSCs

**Figure 1.**
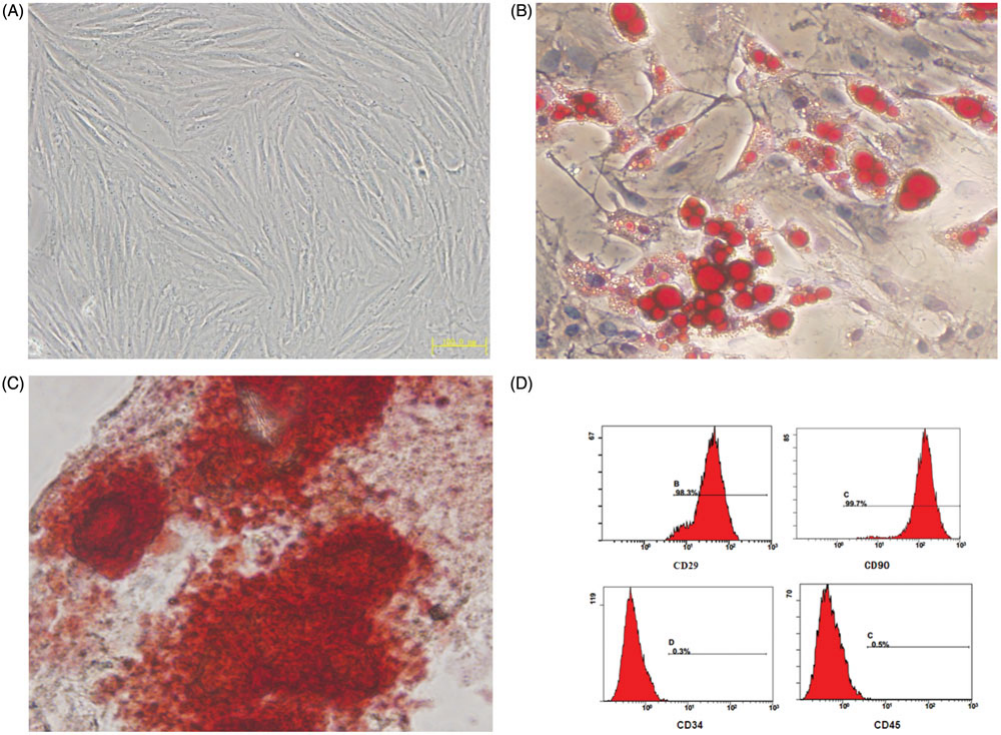
(A) The third generation of BMSCs. (B) Adipogenic differentiation of the BMSCs. (C) Osteogenic differentiation of the BMSCs. (D) Fluorescence-activated cell immunophenotyping analysis of BMSCs.

Morphology of BMSCs was similar to fibroblasts, and their collection in the third generation was just like whirlpool (Figure 1(A)). Adipogenic (Figure 1(B)) and osteogenic (Figure 1(C)) differentiation had been successfully induced, and the Flow cytometry assay (Figure 1(D)) had shown the specific surface molecular markers of BMSCs, both of which indicated that the cultivated cells were rightly BMSCs. Cells from the third to the fifth passages (P_3_–P_5_ cells) were used for gene transfection and animals injection.

### Detection after transfection

**Figure 2.**
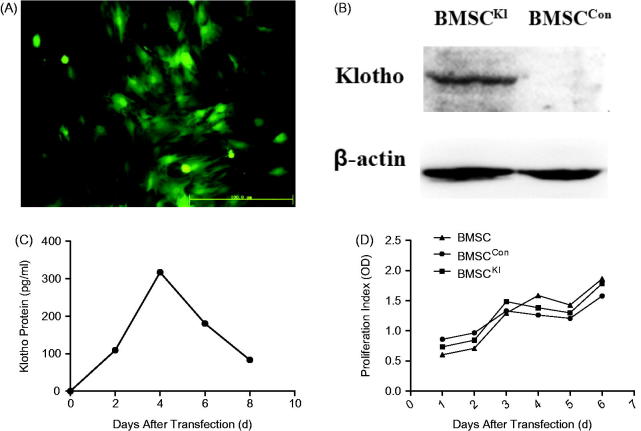
(A) Fluorescence microscope image of BMSCs 48h after transfection. (B) Western blot of Klotho protein in BMSCs after transfection. (C) Secretion curve of Klotho protein expressed by BMSC^Kl^. (D) Proliferation activity of BMSCs.

Fluorescence microscopy has indicated that the expression of Klotho was highest when BMSCs had been transfected by adenovirus for 48 h (Figure 2(A)). Western blot showed that BMSC^Kl^ could express Klotho protein, but BMSC^Con^ could not (Figure 2(B)). This difference was also detected by ELISA which showed that the express was highest on the fourth day after transfection (Figure 2(C)). CCK-8 assay suggested that the proliferation activity of BMSC^Con^ and BMSC^Kl^ were similar with BMSCs within 1 week after transfection (Figure 2(D)).

### Klotho protects BMSCs against RIRI induced-toxicities in vitro

**Figure 3.**
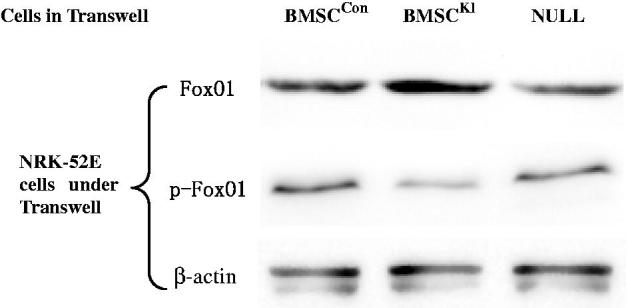
Western Blot for FoxO1 and p-FoxO1 in NRK-52E cells after cocultured with different BMSCs.

Western Blot (Figure 3) showed that after cocultured with BMSC^Kl^, NRK-52E had higher FoxO1 but lower p-FoxO1 expression than that incubated with BMSC^Con^. The result indicated Klotho protein secreted by BMSC^Kl^ might be involved in the regulation of FoxO1 phosphorylation in NRK-52E cells and then promoting the synthesis of SOD in cells, which could improve the ability of antioxidative stress in tubular cells.

### Klotho improves efficacy of BMSCs transplantation in rat model of RIRI

**Figure 4.**
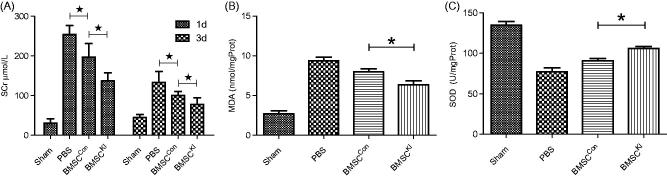
(A) Serum creatinine of each group at 24h and 3d after operation. (B) MDA in renal tissue of each group. (C) SOD in renal tissue of each group. *p < 0.05.

SCr levels of BMSC^Kl^ group were significantly lower than that of BMSC^Con^ group both at 24 h and 3 d after operation, which indicated the BMSC^Kl^ had more strong protective effect than BMSC^Con^ on RIRI (Figure 4(A)). As a metabolic injury index, MDA in homogenated renal tissue of both BMSC^Con^ and BMSC^Kl^ groups were significantly lower than the PBS group, which suggested that BMSCs can reduce the degree of tissue damage after RIRI. Furthermore, MDA of BMSC^Kl^ group was significantly lower than that in BMSC^Con^ group, which showed that BMSC^Kl^ could reduce tissue damage obviously (Figure 4(B)). As a metabolic protective index, SOD were higher in BMSC^Kl^ and BMSC^Con^ groups than those in sham operation group, while SOD value of BMSC^Kl^ group was significantly higher than that in BMSC^Con^ group (Figure 4(C)). The results showed that both BMSC^Kl^ and BMSC^Con^ groups have protective effects on RIRI renal tissue, but the kidney of BMSC^Kl^ group had a stronger ability of antioxidant stress.

### Pathomorphology comparison

**Figure 5.**
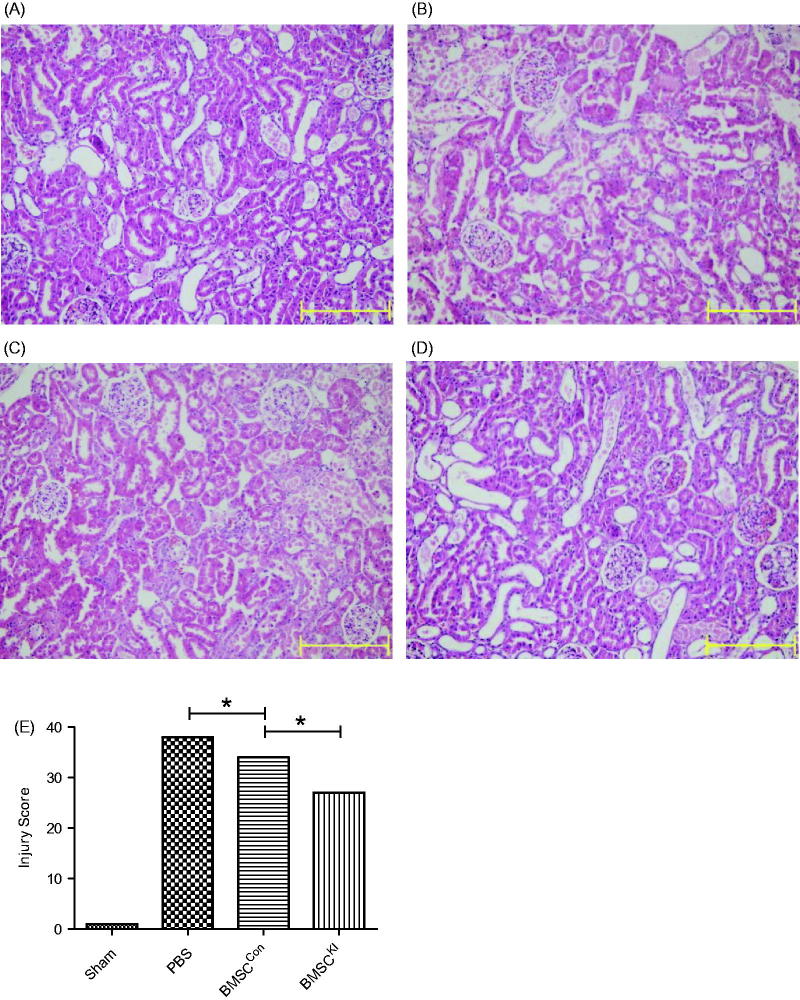
HE staining images 24h after reperfusion. (A) Sham group, (B) PBS group, (C): BMSC^Con^ group, (D) BMSC^Kl^ group, (E) injury score under HE staining of each group. *p < 0.05.

**Figure 6.**
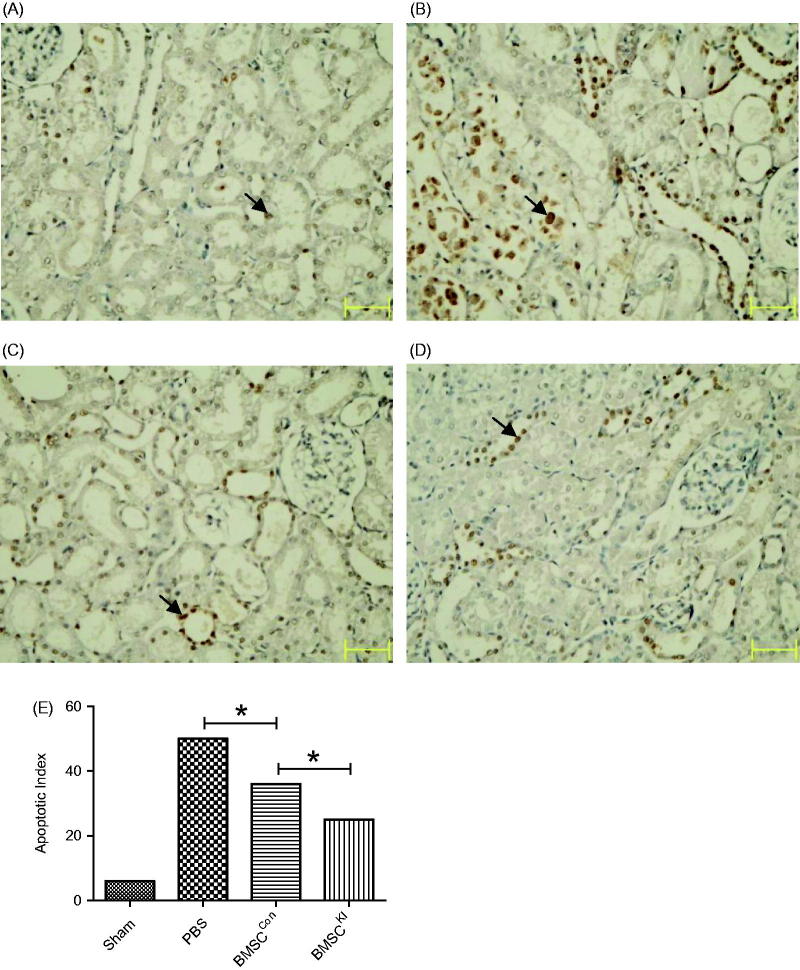
TUNEL cell apoptosis staining 24 h after reperfusion (arrow refers to apoptotic cells). (A) Sham group, (B) PBS group, (C) BMSC^Con^ group, (D) BMSC^Kl^ group, (E) apoptosis Index of TUNEL for each group. *p < 0.05.

Renal tubular injury of BMSC^Kl^ and BMSC^Con^ groups were more obvious than sham operation group but slighter than PBS group at 24 h after RIRI, while tissue damage of BMSC^Kl^ group was slighter than BMSC^Con^ group (Figure 5). TUNEL staining showed the apoptosis index in BMSC^Kl^ group were not only lower than BMSC^Con^ group, but also much more lower than PBS group (Figure 6).

## Discussion

BMSCs transplantation for RIRI treatment had been widely studied in animal experiments [7–9]. Sadek et al. [10] injected BMSCs into RIRI model of America rat by intravenous. They found renal function of treatment group was better than the control group both at 24 h and 72 h after operation. HE, PeriodicAcid-Schiff stain and proliferating cell nuclear antigen (PCNA) staining showed slighter pathological damage in the treatment group. Zhao et al. [11] had injected BMSCs directly into renal parenchyma of RIRI rats, and found IL-6 and TNF-α in renal tissue were lower while vascular endothelial growth factor was higher than the control group. Kwon et al. [12] found that BMSCs could repair endotheliocyte of capillaries around the renal tubular, improved microcirculation of the kidney after RIRI and promoted the recovery of renal function. Iwai et al. [13] found after BMSCs were affused into renal allograft of donation after cardiac death (DCD), not only the renal function ameliorated, but also the survival rate of recipients was improved.

However, *in vivo* survival and repair ability of BMSCs were still need to be improved. Gene transfection was a common technique to enhance these abilities. Qi et al. [14] had found that ‘survivin’ gene transfection could improve BMSCs survival rate *in vivo*. Compared with the control BMSCs, transfected BMSCs can furthermore made the serum SCr and BUN levels of RIRI rats decreasing, the pathological damage of renal tissue improved, and the expression of renal tissue protective factor such as hepatocyte growth factor (HGF) and bFGF increasing. Cao et al. [15] injected CXCR4-BMSC, which can overexpress chemokine receptor 4 (CXCR4), into RIRI rats and found the renal function as well as the renal pathological damage were improved compared with empty-vector group. Fang et al. [16] had transfected human bone morphogenesis protein (hBMP-7) gene into BMSCs to form hBMP-7-BMSC, which were affused to RIRI rabbit through renal artery. They found renal tissue of hBMP-7-BMSC group had higher SOD and Bcl-2, but lower MDA and Bax than BMSC^Con^ group, which showed that after BMSCs transfected with hBMP-7 gene could further enhance renal tissue regeneration ability. Chen et al. [17] found that BMSCs with HGF overexpression could improve the renal function of RIRI rats. There were more PCNA positive cells, fewer tube cast, and lower expression of caspase-3 and IL-1β mRNA in HGF- BMSC group’s renal tissue than that of BMSC^Con^ group.

Klotho protein, which can regulate the apoptosis and mineral metabolism, plays an important role in the pathophysiology of RIRI. Klotho protein deficiency could aggravate tissue damage caused by RIRI, and Klotho protein supplement could alleviate the renal injury [14,18]. Sugiura et al. [19] had directly injected adenovirus with Klotho gene into mouse kidney with RIRI. They found that while the expression of Klotho protein recovered, the increased SCr and renal damage morphology also be alleviated. In addition, overexpression of Klotho can alleviate acute renal injury caused by cisplatin and renal fibrosis caused by unilateral ureteral obstruction [20]. We transformed the Klotho gene into BMSCs and injected BMSC^Kl^ to rat RIRI model. By detecting renal function, kidney tissue oxidative metabolism, renal tissue pathology, and other indicators, we found that BMSC^Kl^ had a stronger ability to repair RIRI than BMSC^Con^.

The key challenge of BMSCs therapy was how to transfuse maximum exogenous cells to the site of target organ, so the route of administration was very important. Most previous studies injected BMSCs via intravenous, which was convenient and easy to operate. However, researchers had recently found most BMSCs were blocked in lung after intravenous injection [21], few of them could reach to liver and kidney. Study of Zou et al. [22] showed that microbubbles (MVs) released by BMSCs could go through pulmonary circulation to the IRI kidney and played a role in recovery. Nevertheless, we still hope to maximize the amount of therapeutic BMSCs into the renal tissue. Therefore, drawing method from Togel et al. [23], we injected BMSCs via the carotid artery, so that BMSCs could enter into the blood flow of aorta and renal artery. Despite not all BMSCs infused into renal artery, the number of BMSCs into RIRI kidney was significantly increased comparing with intravenous injection.

The main pathological manifestation of RIRI was inflammatory reaction, which would decrease the sensibility of kidney microcirculation to vasodilators. With the ability of inflammatory chemotaxis, BMSCs was expected to become an ideal preparation to alleviate RIRI related inflammation [24]. Comparing with bone marrow hematopoietic stem cells, BMSCs have poor differentiation but stronger paracrine function, which were found to be just the main mechanism to relieve RIRI [25–27]. Gene engineering technology could give BMSCs new secretion characteristics, which would enhance BMSCs paracrine ability in order to improve the effect of RIRI recovery.

Mammalian members of the FoxO class of transcription factors are implicated in the regulation of oxidative stress [28], and FoxO1 was found to be a regulator when Jin et al. [29] investigated the role of Klotho on Tac-induced renal injury. In this study, we transferred BMSCs with adenovirus carring Klotho gene, and found that BMSC^Kl^ has stronger ability to cure RIRI than BMSC^Con^. Koltho protein had been found to inhibit the oxidative stress, however, the specific mechanism had not been elucidated in previous reports [30]. By using the cell coculture model, we found Klotho protein secreted by BMSC^Kl^ could downregulate the expression of p-FoxO1, which made more FoxO1 penetrate into the nuclear and combine with SOD promoter (HO-1) in renal tubular epithelial cells. Then the increasing expression of SOD in renal tubular epithelial cells could inhibition of cellular oxidative stress and reduces apoptosis, which can be found by the MDA level and morphologic changes of renal tissue. Therefore, BMSC^Kl^ could inhibit cellular oxidative stress and reduce apoptosis to alleviate RIRI and reduce renal damage via regulation of p-FoxO1 and FoxO1 expression.

In conclusion, BMSCs transfected with Koltho gene had stronger repair ability for RIRI kidney than BMSC^Con^. Future research with large animal model will further confirm its repair advantage. In the future, BMSC^Kl^ might be clinically used in the preservation of DCD kidney and promote the recovery rate of renal function.
